# Iatrol as an Antiseptic

**Published:** 1894-01

**Authors:** D. Eugene Black

**Affiliations:** Buffalo, N. Y.


### IATROL AS AN ANTISEPTIC.

By Dr. D. Eugene Black.

Speaking of antiseptic agents as a rule, I believe I have tried nearly all remedies for an incised wound in the region of the pastern in a coach horse owned by a gentleman in this city, caused by a barb-wire fence blowing down in a storm, and the horse having become entangled in it.

### HISTORY OF THE CASE.

When I was first called to look at the horse he was in a weak state, having bled profusely, and being unattended for twelve hours or more naturally had suffered more or less from hæmorrhage. I bandaged wound with oakum and perchloride of iron until he could be removed to this city, a distance of seven or eight miles, which I was obliged to lead him, having no ambulance handy. On arriving at stable I ordered wound thoroughly washed with a weak solution of carbolic acid 1–1000, and proceeded to stitch wound with catgut sutures, using the interrupted stitch. The horse was very unruly, and after bandaging wound left animal for the night.

In the morning on visiting I was not a little chagrined and discouraged to find that the continued fretting and worrying of animal had torn all the stitches out and had left the foot in a bad condition. I then gave up the idea of healing by first intention and concluded to allow wound to heal by granulation.

Having first cut away all lacerated flesh and common integument, I dressed the wound with iodoform gauze, covering all with a solution of fluid collodion.

The evening of same day again examined wound and found it looking very unsatisfactory, having formed proud flesh and the care did not at all look favorable. To make a long story short, I had wound cleansed daily with carbolized water and dressed with iodoform powder covered with cotton (medicated) for about three weeks, when it still showed no signs of healing.

I then tried iatrol, which proved far superior to iodoform without having the strong odor of the former.

I used the same three times daily, blown on wound with spray blower, and was pleased to note in three days a marked improvement. In just two weeks from the time I began applying iatrol a cicatrix began to form, and to-day the animal is sound and working.

I can recommend this agent for all wounds and foul-smelling abscesses, and think it deserves a further trial in veterinary surgery.

Buffalo, N. Y.

